# Genetic variants of *ABCC8* and clinical manifestations in eight Chinese children with hyperinsulinemic hypoglycemia

**DOI:** 10.1186/s12902-023-01527-8

**Published:** 2024-01-12

**Authors:** Guoying Chang, Lingwen Ying, Qianwen Zhang, Biyun Feng, Ruen Yao, Yu Ding, Juan Li, Xiaodong Huang, Yongnian Shen, Tingting Yu, Jian Wang, Xiumin Wang

**Affiliations:** 1 Department of Endocrinology and Metabolism, Shanghai Children’s Medical Center, School of Medicine, Shanghai Jiao Tong University, 1678 Dongfang Road, 200127 Shanghai, China; 2 Department of Medical Genetics and Molecular Diagnostics laboratory, Shanghai Children’s Medical Center, School of Medicine, Shanghai Jiao Tong University, 200127 Shanghai, China; 3 International Peace Maternity and Child Health Hospital, Shanghai Jiao Tong University School of Medicine, 200030 Shanghai, China

**Keywords:** Hyperinsulinemic hypoglycemia, *ABCC8*, Clinical presentation, Gene abnormalities, Therapy

## Abstract

**Background:**

*ABCC8* variants can cause hyperinsulinemia by activating or deactivating gene expression. This study used targeted exon sequencing to investigate genetic variants of *ABCC8* and the associated phenotypic features in Chinese patients with hyperinsulinemic hypoglycemia (HH).

**Methods:**

We enrolled eight Chinese children with HH and analyzed their clinical characteristics, laboratory results, and genetic variations.

**Results:**

The age at presentation among the patients ranged from neonates to 0.6 years old, and the age at diagnosis ranged from 1 month to 5 years, with an average of 1.3 ± 0.7 years. Among these patients, three presented with seizures, and five with hypoglycemia. One patient (Patient 7) also had microcephaly. All eight patients exhibited *ABCC8* abnormalities, including six missense mutations (c. 2521 C > G, c. 3784G > A, c. 4478G > A, c. 4532T > C, c. 2669T > C, and c. 331G > A), two deletion-insertion mutations (c. 3126_3129delinsTC and c. 3124_3126delins13), and one splicing mutation (c. 1332 + 2T > C). Two of these mutations (c. 3126_3129delinsTC and c. 4532T > C) are novel. Six variations were paternal, two were maternal, and one was de novo. Three patients responded to diazoxide and one patient responded to octreotide treatment. All there patients had diazoxide withdrawal with age. Two patients (patients 3 and 7) were unresponsive to both diazoxide and octreotide and had mental retardation.

**Conclusions:**

Gene analysis can aid in the classification, treatment, and prognosis of children with HH. In this study, the identification of seven known and two novel variants in the *ABCC8* gene further enriched the variation spectrum of the gene.

## Introduction

Hyper-insulinemic hypoglycemia (HH) is the most common cause of persistent hypoglycemia in infants and children and refers to a heterogeneous condition caused by unregulated insulin secretion [1]. Generally, HH presents as fasting hypoglycemia, however, it can also manifest as postprandial, protein/leucine loading-induced, or exercise-induced hypoglycemia. Although HH typically presents in the neonatal period, it can also occur in infancy, childhood, and adulthood with varying severity and etiology [2, 3]. However, owing to the higher glucose consumption rate of neonates and infants, they have a considerably higher risk of permanent brain damage. Therefore, promptly diagnosing and managing HH are important to prevent neurological complications, such as cerebral palsy, epilepsy, neurodevelopmental deficits, and death.

HH can be transient due to various risk factors, or it can be permanent and inherited due to mutations in key genes involved in insulin secretion, such as congenital hyperinsulinism (CHI) [4, 5]. CHI is the most severe form of HH, and mutations in 12 genes have been reported to date [1]. Mutations in the ABCC8 gene, which encodes the SUR1 subunits of pancreatic β-cell K_ATP_ channel, are one of the most common causes of CHI [6–8]. The SUR1 subunit regulates the activity of Kir6.2 proteins and functions as the binding site for the K_ATP_ channel opener (diazoxide) and sulfonylureas, thereby transducing metabolic signals generated by glucose metabolism to regulate insulin secretion [9, 10]. Recessive inactivating mutations of ABCC8 (loss of function) can inhibit the trafficking of SUR1 to the plasma membrane or channel activity, causing medically unresponsive diffuse CHI that may require near-total pancreatectomy [11]. Dominant inactivating mutations in ABCC8 typically cause milder forms of CHI that are responsive to diazoxide.

In this study, we evaluated the clinical and genetic characteristics of eight Chinese children with HH. In line with previous findings, we confirmed the connection between mutations of ABCC8 and CHI and identified two novel mutations among the nine identified. Despite the children inheriting genetic mutations from their parents, their parents did not exhibit any clinical symptoms. Three patients responded to diazoxide, and one responded to octreotide. All patients had good prognosis with respect to ages.

## Methods

### Patients and clinical data collection

The documented medical history of each patient, including birth status, growth, development, past illness, phenotypes, and family history, was preserved for use in this study. Both routine and specific biochemical tests, including routine blood and urine test, alanine aminotransferase (ALT), aspartate aminotransferase (AST), creatine kinase (CK), CK-MB, lactate dehydrogenase (LDH), creatine (Cr), electrolytes, glycated hemoglobin A_1c_ (HbA_1c_), fasting plasma glucose (FPG), and fasting C-peptide (FCP) were performed for each patient. The levels of thyroid hormones, insulin-like growth factor-1(IGF-1), and adrenocorticotropic hormone (ACTH) were also evaluated. The FPG levels were assayed using the glucose oxidase method. HbA_1c_ levels were measured using high-performance liquid chromatography with a VARIANT II Hemoglobin A_1c_ analyzer (Bio-Rad Laboratories, Hercules, CA, USA). C-peptide levels were measured electrochemically (Cobas e 601 Automated Biochemical Analyzer; ROCHE Inc., Basel, Switzerland). Thyroid hormone, ACTH, and IGF-1 indices were determined using a chemiluminescence immunoassay (thyroid hormones and ACTH: CL-8000I; Mindray, Chinese; IGF-1: IMMULITE 2000Xpi; Siemens, Germany). The remaining laboratory indicators were measured using standard methods. Biochemical indicators, particularly blood samples of fasting blood glucose and C-peptide levels, were collected 3–4 h after the latest feeding and immediately before feeding in the morning. Cranial MRI or CT was conducted for all patients except patients 3, 6, and 7 (parents refused due to personal considerations).

Ethical approval was obtained from the ethics committee of the Shanghai Children’s Medical Center, and informed consent was obtained from all the individual participants and their parents or legal guardians included in the study.

### Genetic analysis

Genomic DNA was extracted from peripheral blood samples of each patient and their parents using a QIAamp Blood DNA Mini Kit® (Qiagen GmbH, Hilden, Germany). Targeted next-generation sequencing and data analysis were performed as previously described [12]. All suspected variants were confirmed using Sanger sequencing and validated through parental testing. Manual classification of the variants was performed using the American College of Medical Genetics and Genomics. The potential pathogenicity of novel missense variants was determined using three in silico prediction methods: PolyPhen-2 (http://genetics.bwh.harvard.edu/pph2/), PROVEAN (http://provean.jcvi.org/genome_submit_2.php?species?human), and MutationTaster (http://www.mutationtaster.org/ChrPos. html).

### Diagnose of HH

Patients meeting the following criteria: (1) plasma glucose < 3.0 mmol/L, (2) detectable serum insulin and/or serum C-peptide, (3) suppressed/low β-hydroxybutyrate and acetoacetate, (4) suppressed/low serum free fatty acid, (5) increased requirement of glucose infusion rate (> 8 mg/kg/min), and (6) positive glycemic (> 1.5 mmol/L) response to intramuscular/intravenous glucagon may diagnose with HH. In addition, patients who met one of the aforementioned criteria and had identified causes of acquired HH or a mutation in one of the known causative genes were diagnosed with HH.

### Statistical analysis

All statistical analyses were performed using SPSS (version 24.0; SPSS, Inc., Chicago, IL, USA). Data are presented as the mean ± standard error of the mean (SEM) for continuous variable and as numbers (percentages) for categorical variables.

## Results

### Clinical description and laboratory results for the eight enrolled patients

We analyze the phenotypes and genotypes of eight Chinese children with HH from eight families without consanguineous marriages. The cohort comprised five males and three females with a gestational age of 38.8 ± 0.4 weeks and a birth weight of 4064 ± 272 g. Of these patients, 62.5% (5/8) were classified as large for gestational age (LGA). The age range at presentation was from neonates to 0.5 years old, and the age range at diagnosis was from 1 month to 5 years with an average of 1.3 ± 0.7 years. Of the eight patients, three were referred to our clinic due to seizures (Patients 1, 3, and 7), and five for hypoglycemia (Patients 2, 4, 5, 6, and 8). Patient 7 had microcephaly. Among the patients, the mother of patient 4 alone had gestational diabetes mellitus (GDM) and hypothyroidism, whereas the mother of patient 5 had GDM alone. The remaining patients had no relevant family history. The clinical data for the patients are summarized in Table 1.

**Table 1:** Clinical features of 8 patients with HIH

Patient	Gender	GA (wk)	BW(g)	Age of onset	Age at diagnosis	Current age	Initial presentation	Family history	Treatment and long-term follow-up
P1	Male	38	3300	3 days	0.5 y	5.2 y	seizures	Negative	Diet and antiepileptic drugs, only once hypoglycemia occurred.
P2	Male	39	4000	1 days	0.4 y	1.3 y	hypoglycemia occurred within 24 h after birth	Negative	diazoxide-unresponsive; received octreotide treatment, His glucose level was normal.
P3	Male	38	4500	0.5 y	3.5 y	8.2 y	seizures	Negative	antiepileptic drugs treatment.; Unresponsive to diazoxide and octreotide; glucose supplement if hypoglycemia occurred. And he had mental retardation.
P4	Female	38	5460	1 days	0.1 y	3 y	hypoglycemia occurred within 24 h after birth	Her mother had GDM and Hypothyroidism, and received metformin and Levothyroxine treatment.	diazoxide-responsive, and discontinuation for 1 years, her glucose level was normal
P5	Female	41	4600	10 days	0.1 y	5 y	hypoglycemia	Her mother had GDM and received insulin treatment.	diazoxide-responsive, and discontinuation for 2 years, no hypoglycemia occurred.
P6	Female	39	4000	1 days	0.1 y	4.2 y	hypoglycemia	Negative	diazoxide-responsive, and she received diazoxide treatment till 2 years. No hypoglycemia occurred.
P7	Male	39	3200	1 days	5 y	7 y	recurrent seizures and microcephaly	Negative	Diazoxide and octreotide unresponsive, and hypoglycemia occurred frequently even after pancreatectomy; he had mental retardation.
P8	Male	38	3450	0.6 y	0.6 y	7.6 y	hypoglycemia	Negative	Diet treatment, no hypoglycemia occurred.

GA: gestational age; BW: body weight; GDM: gestational diabetes mellitus

During the hypoglycemic episodes, the lowest recorded blood glucose level was 1.86 ± 0.20 mmol/L. The serum insulin level was 17.6 ± 8.7 µIU/mL (ranging from 5.9 to 77.8 µIU/mL, normal range: 2.3−11.8 µIU/mL), and the c-peptide level was 4.3 ± 1.3 ng/ml (ranging from 0.69 to 10.80 nmol/L; normal range: 0.3−1.6 nmol/L). Urine ketone levels were negative. Routine and specific biochemical tests, including kidney and liver function, and CK, CK-MB, and HbA_1c_ levels were normal. Levels of endocrine hormone, including thyroid hormones, IGF-1, cortisol, and ACTH, were also within the normal range. Abdominal MRI of Patient 5 showed the full pancreas, and cranial MRI revealed deep frontotemporal sulci on both sides. The cranial MRI of Patient 4 showed adenohypophysis hyperplasia, whereas the abdominal and cranial MRI of Patients 1, 2, and 8 were normal. A summary of these laboratory and imaging results is shown in Table 2.

**Table 2:** Laboratory, imaging findings and molecular analysis of 8 patients with HIH

Patient	Glucose (mmol/L)	Insulin (µIU/mL)	C-peptide (nmol/L)	Urine acetone	Imaging finding	ABCC8 abnormality	Inheritance
P1	1.6	6.2	1.02	Negative	Abdominal and cranial MRI was normal.	c. 3126_3129delinsTC, p. Leu1043Hisfs*70	Paternal
P2	0.8	8.8	0.69	Negative	Abdominal and cranial MRI was normal.	c. 2521 C > G, p. Arg841Gly	Paternal
P3	2.5	5.6	2.84	Negative	NA	c. 3784G > A, p. Ala1262Thr	Paternal
P4	1.9	17.9	10.8	Negative	Adenohypophysis hyperplasia	c. 1332 + 2T > C; c. 4478G > A, p. Arg1493Gln	Paternal Maternal
P5	1.9	77.8	8.6	Negative	Full pancreas; deep frontotemporal sulci on both sides	c. 4532T > C, p. Ile1511Thr	Maternal
P6	2.3	5.9	5.8	Negative	NA	c. 2669T > C, p. Leu890Pro	De novo
P7	1.5	12.6	2.4	Negative	NA	c. 3124_3126delins13, p. Thr1042Glnfs*75	Paternal
P8	2.4	6.2	2.3	Negative	Abdominal and cranial MRI was normal.	c.331G > A, p. Gly111Arg	Paternal

*Note*: The glucose level was the minimum value detected, and insulin levels were tested during hypoglycemia. NA, not available

### Identification of *ABCC8* gene abnormality

**Fig. 1:**
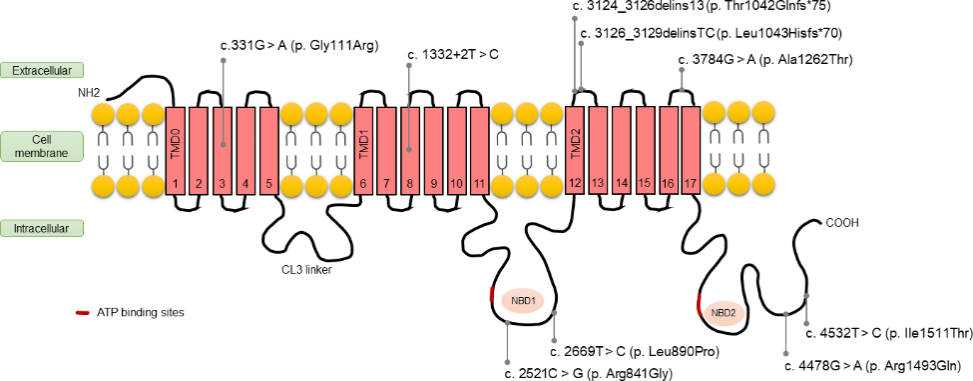
Distribution schematic of the transmembrane topology of the 9 identified variants of ABCC8 gene. The transmembrane domains (TMD) include TMD0, TMD1, and TMD2. The nucleotide-binding domains (NBD) are indicated by NBD1 and NBD2, and the cytosolic loops 3 is between TMD0 and TMD1, respectively. The red bar refers to the ATP binding sites

Table 2; Fig. 1 presents the results of genetic abnormality analysis, which identified seven known variations and two novel variations in *ABCC8* gene in the study. Monoallelic mutation were observed in seven cases, whereas one case had compound heterozygosity. The variants included six missense mutations (c. 2521 C > G, c. 3784G > A, c. 4478G > A, c. 4532T > C, c. 2669T > C, and c. 331G > A), two deletion-insertion mutations (c. 3126_3129delinsTC and c. 3124_3126delins13), and one splicing mutation (c. 1332 + 2T > C). Two of these mutations (c. 3126_3129delinsTC and c. 4532T > C) are novel. Among the variations, 62.5% (5/8) were of paternal origin, two were of maternal origin, and one was de novo. Parents carrying the same variations did not present any clinical symptoms.

We conducted a more detailed analysis of the mutation sites in the eight patients. As illustrated in Fig. 1, two of the mutations were located in the transmembrane domain, whereas three were found in the extracellular topological domain. The remaining four mutations were located in cytoplasmic or intracellular topological domain linked to nucleotide-binding domains (NBD).

### Treatment and long-term follow-up

The effectiveness of the different treatments in these patients was evaluated. Among these patients, six (Patients 2, 3, 4, 5, 6 and 7; 75%) received diazoxide treatment, and 50.0% of those (Patients 4, 5, and 6) were found to be responsive. Patient 1 was treated with a combination of diet and antiepileptic drugs, which resulted in only one instance of hypoglycemia. Patient 8 did not receive any specific drug treatment and remained hypoglycemia-free. Patient 2 received octreotide treatment, which successfully normalized their glucose levels. Patients 3 and 7 were unresponsive to diazoxide and octreotide and had mental retardation. However, all patients who responded to diazoxide were able to withdraw from treatment with age without experiencing hypoglycemia.

## Discussion

According to recent reports, diseases caused by ABCC8 mutations fall into the following categories: HH, permanent neonatal diabetes mellitus, transient neonatal diabetes mellitus, and pulmonary arterial hypertension. More than 400 ABCC8 mutations have been reported to cause HH. At least 14 ABCC8 mutations have been identified in patients with permanent neonatal diabetes mellitus [13]. Other ABCC8 gene mutations that have relatively mild effects on K_ATP_ channel function compared to those seen in permanent neonatal diabetes mellitus cause transient neonatal diabetes mellitus [14]. Moreover, an ABCC8 mutation was detected in one patient with pulmonary arterial hypertension [15].

There were three forms of HH, namely, sporadic, autosomal recessive, and autosomal dominant [12]. Patients with sporadic forms usually have no family history, present with moderate/severe episode of hypoglycemia/hyperinsulinemia in the first days to weeks of life, and generally respond poorly to medical management but have excellent prognosis after partial or > 95% pancreatectomy [16]. Patients with the recessive form usually have severe clinical presentation in infancy, with symptoms and signs secondary to hypoglycemia and hyperinsulinemia. These patients frequently do not respond to medical management and require surgical intervention in the form of near-total pancreatectomy [16]. Autosomal dominant HH patients usually have a relatively mild clinical presentation than the autosomal recessive form, usually onset at 6–9 months of age or later, and are quite responsive to medical therapy with diazoxide [17].

In the present study, the clinical manifestations and prognosis of eight individuals with *ABCC8* mutation were not identical. Our results showed that most patients had a good prognosis with age, and some had good blood glucose control after drug withdrawal without any hypoglycemic events. However, two patients (Patients 3 and 7) had mental retardation and recurrent episodes of hypoglycemia. We found that both those patients had poor prognosis at onset with seizures, and one of them even had microcephaly. Considering that Patient 1 also had the onset of seizure, but with a better prognosis, unresponsiveness to both diazoxide and octreotide seems to have contributed to the worsened prognosis. The present study demonstrated that, under normal circumstances, ATP levels were relatively low when the blood glucose concentration decreased, which opened the K_ATP_ channel, the consequent K^+^ outflow polarized the pancreatic β-cell membrane, resulting in reduced insulin secretion [18]. Therefore, diazoxide (open the K_ATP_) can be used to treat HH. In addition, octreotide can be used to treat diazoxide-unresponsive HH owing to its inhibitory effect on insulin release [18–20]. Nevertheless, Patient 2 (onset with hypoglycemic), though unresponsive to diazoxide, showed a good prognosis. Thus, timely diagnosis and treatment appear to be important indicators of prognosis.

Additionally, we found a correlation between the mutation sites and the onset and prognosis of the affected children. Patient 8, who had monoallelic missense mutations in the extracellular topological domain, had the latest onset age (6 months) and the best prognosis, with no hypoglycemia occurring when treated with dietary interventions alone. Conversely, Patient 3, with compound heterozygosity, was unresponsive to diazoxide and octreotide and suffered from mental retardation. Patients 4 and 5 had mutations in the NBD2 region, and both presented with onset hypoglycemia, which was diazoxide-responsive; no hypoglycemia occurred after drug withdrawal. Patients 2 and 6 harbored mutations in NBD1. Patient 6 was diazoxide-responsive, whereas Patient 2 was diazoxide-unresponsive but octreotide-responsive. The difference in their responses to drugs may be related to the proximity of the mutation site to the ATP-binding sites.

Notably, Patients 1 and 7 experienced seizures at the onset and had deletion-insertion mutations in the extracellular topological domain that predictably affected the function of the entire NBD2. However, their prognoses differed substantially: Patient 1, who had a novel mutation, only received dietary intervention and antiepileptic drugs, and had only one episode of hypoglycemia, whereas Patient 7 was unresponsive to diazoxide and octreotide and frequently experienced hypoglycemia even after pancreatectomy, leading to mental retardation. We hypothesize that the protein formed after the mutation in Patient 1 may partially replace the function of the NBD2 region, which may explain the relatively good prognosis. In a recent study of 18 nonpancreatectomized patients with ABCC8 variants, 41.7% of patients subsequently progressed to diabetes [21]. Regular follow-up of glucose metabolism after remission is recommended.

Interestingly, most patients in this study had heterozygous mutations, which are usually paternal. However, the father showed no hypoglycemic symptoms. The allelic expression imbalance (AEI) may explain the discrepancy in that both father and his child had the same mutated gene but showed different phenotypes [22]. AEI is defined as the phenomenon in which two alleles of a gene differ in their expression magnitude. Previously, it was believed that the expression of both paternal and maternal haplotypes was balanced and that balanced expression could reduce the recessive deleterious mutations. However, mRNA expression is regulated by several mechanisms, including SNP [23]. Recent findings demonstrated that AEI occurred when the transcription of one allele was selectively silenced or enhanced or when the transcript was selectively degraded post-transcriptionally [24]. Such an allelic imbalance in gene expression may be associate with phenotypic variations among individuals and contribute to human disease. However, whether there is an AEI in the *ABCC8* gene requires further investigation.

## Conclusion

The present study evaluated the genetic variants in *ABCC8* genes in eight children with HH. The results revealed seven known and two novel variations. The clinical manifestations and prognosis of the eight individuals with *ABCC8* mutation were not identical; specifically, most of the patients had a good prognosis with age, and some of them had good blood glucose control after drug withdrawal without any hypoglycemic events. Their parents, who carried the same variation, showed no clinal presentation; an allelic expression imbalance may explain this discrepancy.

## Data Availability

The datasets generated and/or analyzed during the current study are available in the NCBI ClinVar database, with accession number to datasets of SCV003922074 and SCV003922072 (https://www.ncbi.nlm.nih.gov/clinvar/variation/2137006/; https://www.ncbi.nlm.nih.gov/clinvar/variation/2501665/).
